# Analysis of US Household Catastrophic Health Care Expenditures Associated With Chronic Disease, 2008-2018

**DOI:** 10.1001/jamanetworkopen.2022.14923

**Published:** 2022-05-27

**Authors:** Young-Rock Hong, Zhigang Xie, Ryan Suk, Amir Alishahi Tabriz, Kea Turner, Peihua Qiu

**Affiliations:** 1 Department of Health Services Research, Management and Policy, College of Public Health and Health Professions, University of Florida, Gainesville; 2 UF Health Cancer Center, Gainesville, Florida; 3 Department of Management, Policy and Community Health, The University of Texas Health Science Center School of Public Health, Houston; 4 Department of Health Outcomes and Behavior, Moffitt Cancer Center, Tampa, Florida; 5 Department of Oncological Sciences, University of South Florida Morsani College of Medicine, Tampa; 6 Department of Biostatistics, University of Florida, Gainesville

## Abstract

This cross-sectional study evaluates trends in catastrophic health care expenditures associated with chronic diseases in US households from 2008 to 2018.

## Introduction

More than 60% of US adults have at least 1 chronic condition, and 40% live with 2 or more chronic conditions.^1^ Studies have evaluated trends in the prevalence of catastrophic health care expenditures (CHCEs) in US non-elderly adults,^2^ but little is known about these trends among households with members with chronic conditions.

## Methods

This cross-sectional study used pooled data from the 2008-2018 Medical Expenditure Panel Survey (MEPS), which contains information on health care expenditures and sources of payment in the US population. The University of Florida institutional review board deemed this study exempt from review with a waiver of informed consent because data were deidentified and publicly available from the Agency for Healthcare Research and Quality. The study followed the STROBE reporting guideline.

Individual household was the unit of analysis. Out-of-pocket spending included deductibles, co-insurance, co-payments, and other cost-sharing for health care. Premium payments were the total amount households paid for health insurance. Individual expenditures were aggregated at the household level using a unique household identifier. A CHCE was identified when the sum of out-of-pocket and premium payments incurred by all members of the household exceeded 40% of total annual household income.^2^ Chronic condition types were selected based on the MEPS Priority Conditions identified as the most costly.^3^

Using generalized regression models with lognormal distribution, we calculated changes in prevalence of households with CHCEs. Models were adjusted for household size, income, insurance type, census region, age, sex, race and ethnicity, and educational level. Given significant interaction terms, analyses were stratified by household income level and chronic conditions status. Analyses were conducted using SAS, version 9.4, adjusting for MEPS complex survey design. Two-sided *P* < .05 was considered significant. Data were analyzed from July 2021 to April 2022.

## Results

Analyses included 140 301 individuals (52.7% male; mean [SD] age, 49.1 [17.1] years), representing 130 million households (median household size, 2 [IQR, 1-4]). Between 2008 and 2018, the estimated prevalence of households with CHCEs did not change (4.7% [95% CI, 3.8-5.6] vs 4.4% [95% CI, 3.6-5.3]; *P* = .34) (Table). Significant decreases in the prevalence of CHCEs were observed among households with any chronic condition (−1.5%; 95% CI, −2.4 to −0.6; *P* = .002). The decrease was largest among households with members with lung disease (−3.4%; 95% CI, −6.0 to −0.8; *P* = .01) or multimorbidity (−3.3%; 95% CI, −4.9 to −1.7; *P* < .001).

**Table.** Prevalence of Catastrophic Health Care Expenditures Among US Households, 2008-2018^a^

Subpopulation	Catastrophic health care expenditures^b^			*P* value	Annual change, % (95% CI)	*P* value for trend
	2008	2018	Difference			
Overall	4.7 (3.8 to 5.6)	4.4 (3.6 to 5.3)	−0.3 (−0.9 to 0.3)	.34	−1.0 (−2.5 to 0.5)	.17
Total OOP spending, mean (SE), $^c^	4685 (95)	5565 (125)	884 (574 to 1193)	<.001	0.9 (0.5 to 1.2)	<.001
Premium contribution, mean (SE), $^c^	3121 (78)	3897 (112)	776 (508 to 1044)	<.001	1.7 (1.3 to 2.2)	<.001
Chronic condition status						
Without chronic condition	2.2 (1.6 to 2.9)	2.9 (2.2 to 3.5)	0.6 (−0.1 to 1.4)	.10	1.0 (−1.2 to 3.2)	.37
With any chronic condition	5.7 (4.5 to 6.9)	4.2 (3.0 to 5.3)	−1.5 (−2.4 to −0.6)	.002	−3.0 (−4.8 to −1.1)	.002
Chronic condition type						
Heart disease	5.8 (4.4 to 7.3)	3.9 (2.6 to 5.3)	−1.8 (−3.1 to −0.5)	.005	−2.4 (−4.7 to −0.1)	.04
Cancer	6.3 (4.3 to 8.3)	3.4 (1.9 to 4.9)	−2.9 (−4.5 to −1.3)	<.001	−4.8 (−7.8 to −1.7)	.003
Stroke	6.7 (3.9 to 9.4)	4.8 (2.6 to 7.1)	−1.8 (−4.4 to 0.8)	.17	−4.6 (−8.8 to −0.3)	.04
Diabetes	6.2 (4.5 to 7.8)	4.2 (2.9 to 5.5)	−2.0 (−3.5 to −0.4)	.01	−3.9 (−6.9 to −0.8)	.02
Lung disease	5.6 (3.2 to 8.0)	2.2 (0.3 to 4.0)	−3.4 (−6.0 to −0.8)	.01	−7.5 (−12.0 to −2.8)	.002
≥2 Chronic conditions	7.4 (5.4 to 9.3)	4.1 (2.4 to 5.7)	−3.3 (−4.9 to −1.7)	<.001	−5.1 (−7.7 to −2.4)	<.001
Household income level^d^						
Low	10.4 (8.7 to 12.2)	9.9 (8.2 to 11.5)	−0.5 (−2.1 to 1.0)	.48	−1.0 (−2.7 to 0.7)	.25
Middle	3.5 (2.1 to 5.2)	3.7 (2.1 to 5.2)	0.2 (−0.6 to 1.1)	.62	0.8 (−2.6 to 4.3)	.64
High	0.2 (0.0 to 0.6)	0.5 (0.0 to 0.9)	0.3 (−0.1 to 0.7)	.13	9.0 (2.2 to 16.3)	.009
Insurance type						
Any private	3.9 (3.3 to 4.4)	3.9 (3.3 to 4.6)	0.0 (−0.6 to 0.8)	.82	1.1 (−1.0 to 3.2)	.31
Medicaid and other public^e^	3.0 (1.8 to 4.2)	1.7 (0.7 to 2.7)	−1.3 (−2.6 to −0.7)	.01	−4.6 (−8.3 to −0.7)	.02
Medicare	3.1 (1.7 to 4.5)	1.6 (0.7 to 2.5)	−1.5 (−2.9 to −0.0)	.049	−4.4 (−8.5 to 0.0)	.047
Uninsured	2.5 (0.6 to 4.4)	2.1 (0.2 to 4.1)	−0.4 (−1.8 to 1.1)	.63	−3.2 (−8.6 to 2.6)	.27

Abbreviations: FPL, federal poverty level; OOP, out-of-pocket.

^a^ Household was defined based on reporting units indicating a group of persons related by blood, marriage, adoption, or other family association, and health insurance eligibility units including all adults and children under health insurance family plans. Catastrophic health care expenditure was identified when the sum of out-of-pocket and premium payments incurred by all members of the household exceeded 40% of total annual household income. The analysis incorporated Medical Expenditure Panel Survey family-level weight and variance adjustments to produce nationally representative estimates. Estimates were adjusted for household size; income; insurance type; census region; and reference person’s age, sex, self-reported race and ethnicity (Hispanic, non-Hispanic Black, non-Hispanic White, and other [Alaska Native, American Indian, Asian/Pacific Islander, and multiracial]), and educational level (high school or less, some college, college degree, and graduate or higher).

^b^ Data are presented as percentage of expenditures (95% CI) unless otherwise indicated.

^c^ All the expenditures were converted to 2018 USD using the Consumer Price Index from the US Bureau of Labor Statistics.

^d^ Based on the federal poverty guidelines (low income: FPL, <200%; middle income: FPL, 200%-400%; high income: FPL, >400%).

^e^ Includes other state-based programs.

The difference in CHCEs for any chronic condition was greatest among low-income households (annual percent change [APC], −3.0%; 95% CI, −4.6% to −1.1%; *P* = .002) (Figure). Middle-income (APC, −5.5%; 95% CI, −10.2% to −0.6%; *P* = .03) and low-income (APC, −3.3%%; 95% CI, −6.1% to −0.5%; *P* = .02) households with members with multimorbidity had significantly decreased prevalence of CHCEs.

**Figure.**
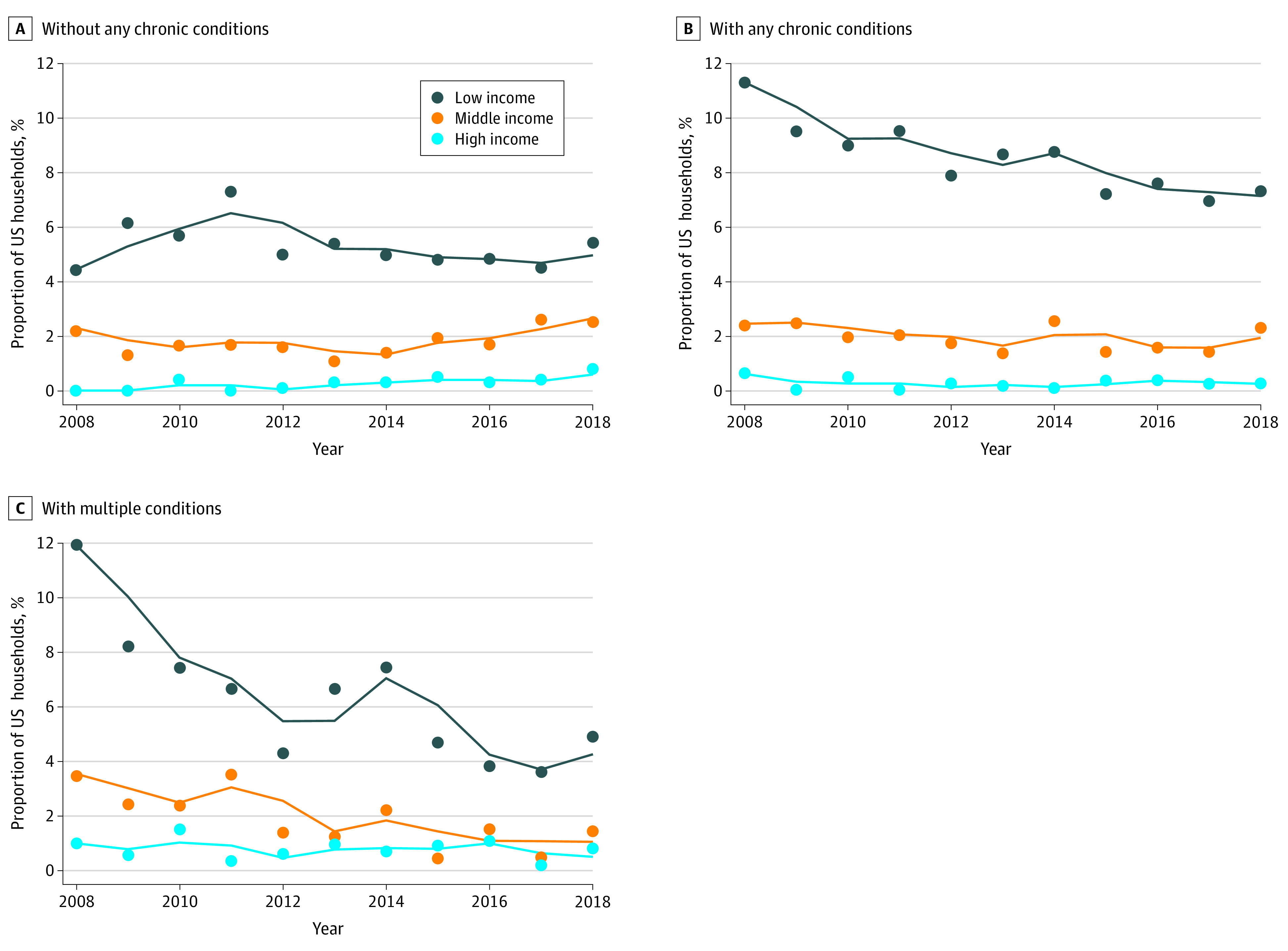
Trends in Catastrophic Health Care Spending Among US Households by Household Income Level and Chronic Condition Status Between 2008 and 2018 Chronic conditions included heart disease, cancer, stroke, diabetes, lung disease, and multimorbidity (≥2 conditions). Household income level was based on the federal poverty guidelines (low income: federal poverty level [FPL], <200%; middle income: FPL, 200%-400%; high income: FPL, >400%).

## Discussion

Between 2008 and 2018, there was a modest decrease in CHCEs among US households with chronic conditions that was greater among households with multimorbidity or low income. Although we did not observe changes from 2010 to 2014 (Patient Protection and Affordable Care Act [ACA] implementation period), provisions of the ACA (mainly coverage expansion) may have been associated with reduced excessive health care spending among households with low income and high disease burden.^4,5^ CHCEs did not significantly change among households with middle or high income; thus, evidence for change in the overall catastrophic health care burden is inconclusive. Previous studies suggested that increases in premium contribution and high-deductible plans may offset the reduction in health care spending associated with the ACA.^6^ We observed higher APC for premium payments than total out-of-pocket spending.

Limitations include the cross-sectional design, which prohibits causal inferences. We could not assess perceived or materialized financial hardship because that information was not available. This study provides insight into CHCEs among US households that may guide future health care policy, but future studies are warranted to examine other aspects of financial burden among individuals with chronic conditions.
